# Ileal intussusception due to heterotopic pancreas in the ileum: a case report

**DOI:** 10.1093/jscr/rjae570

**Published:** 2024-10-07

**Authors:** Francesco Cammarata, Al’ona Yakushkina, Martina Aguzzi, Claudio Guerci, Niccolò Fioritti, Piergiorgio Danelli

**Affiliations:** Luigi Sacco University Hospital, Department of General Surgery, Via G.B. Grassi 74, 20157 Milan, Italy; University of Milan, Department of General Surgery, Via Festa del Perdono 7, 20122 Milan, Italy; Ospedale di Saronno, Department of General Surgery, Piazzale Don Giuseppe Borella 1, 21047 Saronno (VA), Italy; Luigi Sacco University Hospital, Department of General Surgery, Via G.B. Grassi 74, 20157 Milan, Italy; University of Milan, Department of General Surgery, Via Festa del Perdono 7, 20122 Milan, Italy; Luigi Sacco University Hospital, Department of General Surgery, Via G.B. Grassi 74, 20157 Milan, Italy; University of Milan, Department of General Surgery, Via Festa del Perdono 7, 20122 Milan, Italy; University College of London, Department of Cell and Developmental Biology, Gower Street, London, WC1E 6BT, United Kingdom; Luigi Sacco University Hospital, Department of General Surgery, Via G.B. Grassi 74, 20157 Milan, Italy; Department of Biomedical and Clinical Sciences, Luigi Sacco University of Milan, Via G.B. Grassi 74, 20157 Milan, Italy

**Keywords:** heterotopic pancreas, intussusception, pancreas, acute abdomen, case report

## Abstract

Heterotopic pancreas (HP) is a rare condition where pancreatic tissue is found outside its usual location, usually within the gastrointestinal tract. While typically asymptomatic, HP can cause complications like gastrointestinal bleeding and intussusception, especially in adults, posing diagnostic and therapeutic challenges. A 31-year-old male presented with severe abdominal pain, nausea, and vomiting. Initial imaging revealed significant ileal and cecal wall thickening. Despite antibiotic therapy, his condition worsened, necessitating exploratory laparotomy. Intraoperative findings showed ileal intussusception near the cecum, leading to ileocecal resection and ileo-colic anastomosis. Pathological examination confirmed HP as the cause of intussusception. This case underscores the importance of considering HP in adult intussusception. Timely surgical intervention is critical to prevent severe complications. At a two-year follow-up, he remained symptom-free, highlighting the necessity for prompt diagnosis and management.

## Introduction

Heterotopic pancreas (HP) refers to the presence of pancreatic tissue in organs different from the pancreas itself, usually in other portions of the gastrointestinal tract. The real prevalence is unknown as it’s usually asymptomatic, but it may causes different clinical pictures [1]. Due to its rarity, it can pose problems in the diagnostic and therapeutic management. We encountered a case of ileal intussusception as consequence of ileal HP, which represented a very unusual surgical emergency.

## Patient

**Figure 1:**
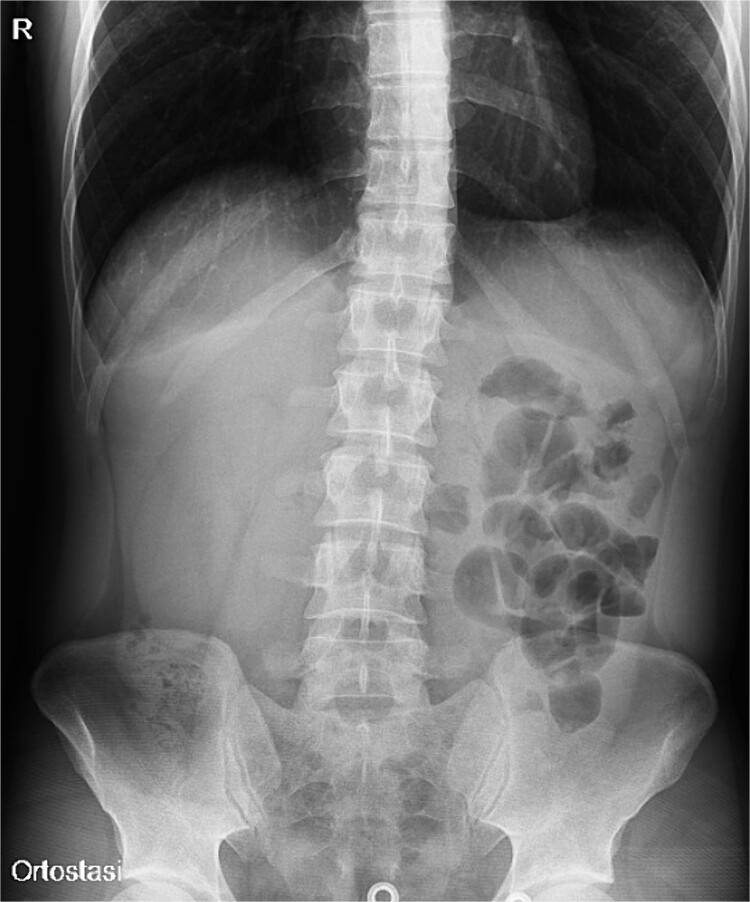
Plain abdomen X-ray showing limited visibility of the colon on the right side with small bowel loops’ distension on the left regions, without free air.

**Figure 2:**
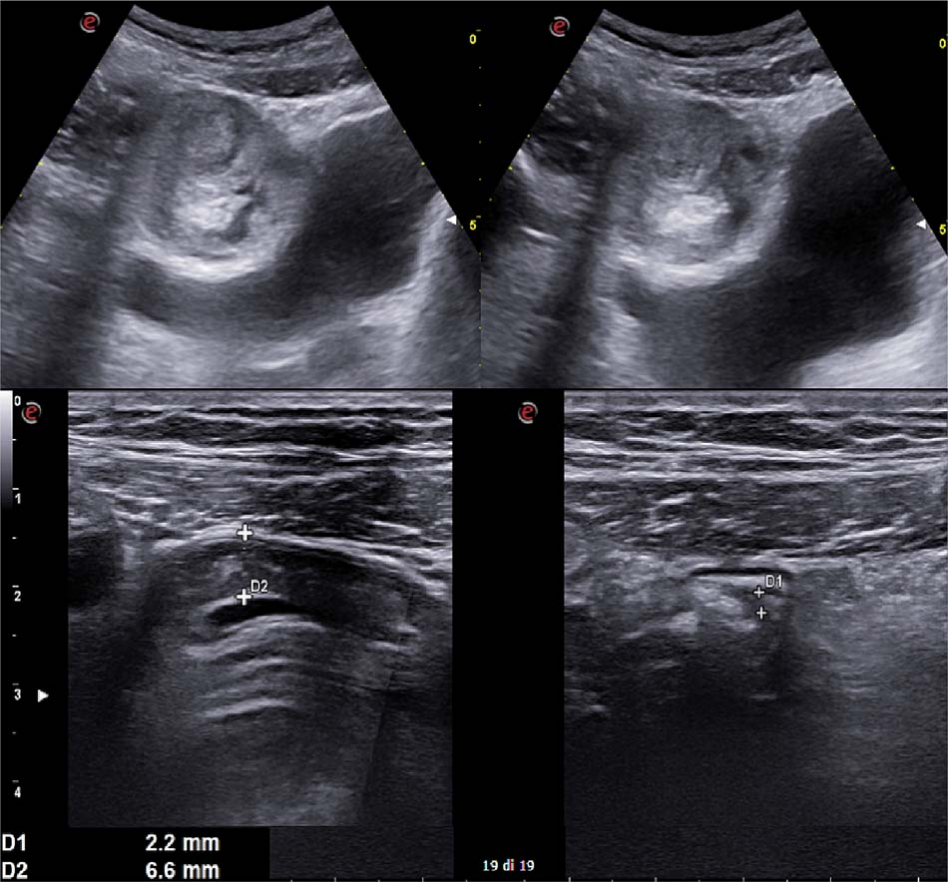
Abdomen ultrasound image displaying visceral distension with a cockade appearance consistent with an inflammatory condition of the cecum and the ileocecal site, showing wall thickening of 6 up to 10 mm and free fluid near the cecum.

**Figure 3:**
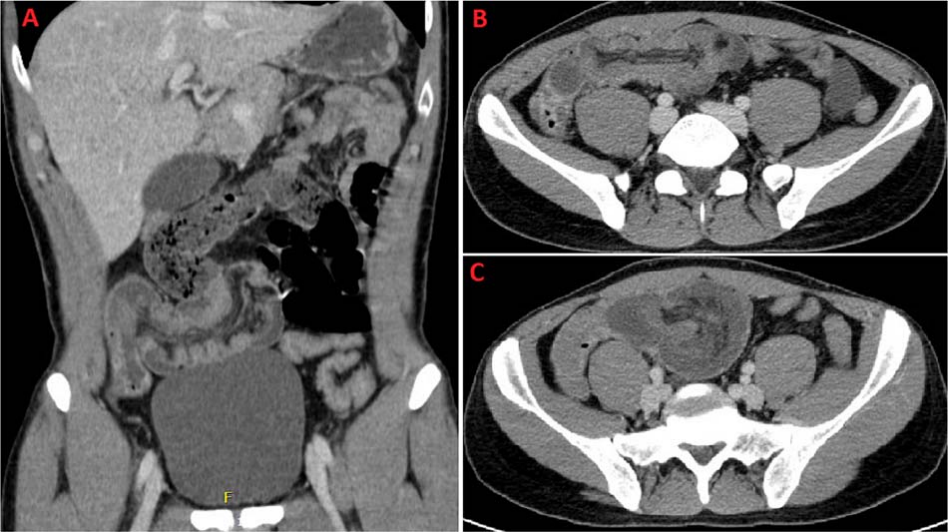
A and B: Coronal and axial sections of contrast-enhanced CT scan confirming wall thickening of the last ileal loop and the cecum up to 20 mm. C: CT imaging performed the following day, displaying increased wall thickening up to 24 mm with the last ileal loop appearing twisted around its mesentery.

A 31-year-old male was complaining episodes of abdominal pain in the umbilical region with nausea. In medical history he underwent an appendectomy. Chronic gastritis was found on esophagogastroduodenoscopy (EGD) and proton-pump inhibitors (PPI) treatment was started, with regression of symptoms. After two months, he took ketoprofen for back pain for two days when a severe abdominal pain in the umbilical and right iliac region started, with nausea and vomit. He had normal vital signs and tenderness in the right lumbar and iliac regions. Blood exams showed leucocytosis (GB 14.120 × 10^6^/ml) with neutrophilia (88%). A plain abdomen X-ray showed absence of representation in the right regions, with small bowel loops’ distension on the left (Fig. 1). An abdomen US displayed the presence of visceral distension with cockade appearance, consistent with an inflammatory condition of the cecum and the ileocecal site, with wall thickening of 6 up to 10 mm and free fluid near the cecum (Fig. 2). Contrast-enhanced CT confirmed wall thickening of the last ileal loop and the cecum up to 20 mm (Fig. 3A and B). Antibiotic therapy was started. The next day the pain worsened and there was an abdominal mass in the right iliac region with rebound tenderness. Blood examination showed further increase in leucocytosis (GB 18560) and a new CT was done. The wall thickening increased up to 24 mm, and the last ileal loop was more hypointense, with a twisted appearance around its mesentery (Fig. 3C). Exploratory laparotomy was performed. During surgery we found an ileal intussusception of the distal ileum near the cecum, and an ileocecal resection with ileo-colic anastomosis was done. On the first post-operatory day he developed haematochezia and acute anaemia, so he underwent reintervention with resection of the anastomosis and ileostomy. The following post-operative course was uneventful.

On pathological examination of 41 cm of ileus and 5 of cecum, they found a 29 cm ileo-ileal intussusception that goes 6 cm near the ileocecal valve. The everted ileum was characterized by haemorrhagic submucosal and mucosal infarction. Near the proximal margin, nodular lesion within the ileal wall was found. It was duro-elastic and grey-pink, with a maximum diameter of 2 cm, consistent with nodular heterotopia of normal pancreatic tissue in the submucosal and the internal half of the muscular layer of the ileum.

After 6 months, he underwent reversal of the stoma and after a two-year follow-up he has no health problems.

## Discussion

HP is defined as pancreatic tissue lacking anatomical or vascular connection to the pancreas. In the majority of reported cases (70%–90%) [2] the occurrence of HP is reported in the gastrointestinal system (stomach, duodenum, jejunum, Meckel’s diverticulum, colon, gallbladder, and liver) [2–4]. However HP has been reported rarely in other anatomical districts as fallopian tubes, mediastinum, spleen, mesentery and umbilicus [5]. While it remains unclear, one of the main hypotheses for its aetiology identifies during the embryological rotation of the foregut: at the moment of the fusion of the dorsal and ventral pancreatic buds, part of the pancreatic tissues separates from the developing pancreas colonizing the digestive tracts [2]. It is alternatively proposed that HP could arise from metaplastic phenomenon occurring during embryological development [1, 2, 4].

A classification system for HP was initially developed by Heinrech in 1909 and later refined in 1973 by Gaspar-Fuentes [4]. It divides HP cases into four main classes: Type-1, composed of typical pancreatic tissue with acini, islet cells, and ducts similar to normal pancreas; Type-2, comprising only pancreatic ducts; Type-3, constituted by acinar tissue only, and Type-4 which solely includes islet cells [4, 6].

The real prevalence of HP is unknown. Prevalence on autoptic findings is between 0.5% and 13.7% [1]. HP cases are largely asymptomatic, thus remaining undiagnosed. Majority of reported HP cases (up to 65.5%) has been identified incidentally [4]. Only 6.9% of the reported HP cases have been diagnosed after investigating secondary complications [4].

While largely asymptomatic, HP is correlated with clinical manifestations typical for the orthotopic pancreas such as pancreatitis, pseudocyst formation and benign and malignant neoplasms [1]. Unspecific symptoms include gastrointestinal bleeding, bowel obstruction, and abdominal pain [7–12]. Malignant transformation of HP has been also identified as an extremely rare event, with only 17 cases reported in literature [13]. Intussusception represents 2% of the described complications of HP. [4]

Intussusception *per se* is more frequent in children than in adults, representing the most common cause of occlusion in children, while accounting for only the 0.1% of adult hospitalizations [14, 15]. In children intussusception is mostly idiopathic, in adults a clinical cause can be identified in up to 90% of the cases. Within those, benign tumours, including HP, account for 21% of colonic intussusception and 40% of small bowel intussusception [14]. Specifically, HP is reported being the pathological leading point for 2% to 12% of total intussusception cases [5].

Because of the high frequency of asymptomatic HP cases, their diagnosis is problematic. Due to the low incidence of specific HP complication, clinicians should be aware of the relatively high prevalence of HP and its meaning in the comprehensive clinical picture. Diagnosis is generally made on histopathology, but capsule endoscopy has been reported being an efficient identification tool for ileal HP while investigating cases of epigastric pain and bloody stool [16].

The treatment is generally necessary for symptomatic or complicated HP cases and it’s dependent on presentation’s severity and localization. For gastrointestinal lesions, resection is usually recommended. Endoscopic mucosal or submucosal resection can be sometimes performed for less severe presentation. However, due to high incidence of complication and lower therapeutic yield of endoscopic techniques, surgical resection should be preferred for asymptomatic lesions > 3 cm, symptomatic ectopic pancreas, biopsied lesions with evidence of premalignancy, or lesions located in the muscularis propria, subserosa, or serosal layers of the GI tract [4, 7, 13, 14].

## Conflict of interest statement

All authors declare that they have no conflict of interest. Informed consent was obtained from the patient being included in the study.

## Funding

None declared.
